# A New Gain Spiral at Work: Relationships between Virtuous Organizational Practices, Psychological Capital, and Well-Being of Workers

**DOI:** 10.3390/ijerph20031823

**Published:** 2023-01-19

**Authors:** Julia Aubouin-Bonnaventure, Evelyne Fouquereau, Hélène Coillot, Fadi-Joseph Lahiani, Séverine Chevalier

**Affiliations:** 1 QualiPsy EE 1901, Psychology Department, University of Tours, 37041 Tours, France; 2 AD Conseil, 92240 Malakoff, France

**Keywords:** virtuous organizational practices, psychological capital, job satisfaction, thriving at work, work–life balance

## Abstract

Identifying antecedents of well-being at work is an active field of research, focusing notably on organizational practices that promote employees’ optimal health. To date, whereas studies have demonstrated that some organizational practices, considered in isolation, are positively associated with indicators of well-being, none tested the joint effect of a bundle of practices on these. Moreover, few studies have examined the psychological mechanisms underlying these relationships. The present study aimed to identify the relationships between virtuous organizational practices, a new psychological integrative construct, and three indicators of workers’ hedonic, eudaimonic, and social well-being, namely job satisfaction, thriving at work, and work–life balance, and to test the mediational role of psychological capital in these relationships. The sample comprised 400 French employees working in non-profit, private, and public organizations. Structural equation modeling confirmed the direct effects of virtuous organizational practices on the three indicators of well-being, and a bootstrapping procedure demonstrated that psychological capital partially mediates these relationships. The results of this study have many practical applications because virtuous organizational practices can easily be implemented and optimized in work organizations to develop the individual resources of workers and, in detail, to promote their psychological well-being. Finally, the contributions of this study, avenues for future research, and limitations are discussed.

## 1. Introduction

Well-being at work has been widely demonstrated to have positive effects for both individuals and organizations, notably in terms of psychological health and performance. At the employee level, well-being is positively related to positive emotions [1], flow at work [2], life satisfaction [1], and work–family enrichment [3], and negatively related to work-related rumination [4], job stress [5], mental distress [1], and burnout [6]. At the organizational level, workers’ well-being is associated with increased performance [7]. For example, in a recent meta-analysis of 45 independent samples with a total of 34,221 participants, [8] found that psychological well-being was positively related to overall, task, and contextual performance. Well-being has also been shown to be related to positive attitudes and behaviors such as organizational commitment [9], organizational citizenship behaviors [10], and trust in the direct leader and in organizational leadership [11]. For this reason, identifying the antecedents of well-being has become an active field of research [12]. For example, studies have shown that work motivation (i.e., individual), authentic leadership, transformational leadership (i.e., managerial), perceived organizational support, and perceived corporate social responsibility (i.e., organizational) are predictors of workers’ job satisfaction and flourishing [13,14,15,16]. In this study, we investigated whether Virtuous Organizational Practices (VOP) are related to the psychological well-being of French employees.

The construct of VOP has recently been defined as “formal organizational practices that focus on employees’ psychological well-being and optimal health” (p. 2) [17]. In positive organizational scholarship, the concept of virtuousness refers to organizational characteristics that promote the optimal functioning of workers [18,19]. More precisely, VOP are an integrative construct (i.e., it brings together a set of practices) based on the American Psychological Association’s (APA) model of Psychologically healthy workplaces, and includes: (1) Practices of Participative Decision-Making (PPDM) (e.g., decision-making meetings, problem-solving groups, and referendums), (2) Work–Life Balance Practices (WLBP) (e.g., flextime, part-time work, and telecommuting), (3) Health and Safety Practices (HSP) (e.g., well-being programs), (4) Recognition Practices (RP) (e.g., bonuses, awards, and improving working conditions), (5) Practices of Career Management (PCM) (e.g., training programs, internal mobility, and promotion), (6) Communication Practices (CP) (e.g., information meetings, employee surveys, and social media), (7) Organizational Justice Practices (OJP) (e.g., transparent salary policy and equal opportunity program), and (8) Social Dialogue Practices (SDP) (e.g., regular collective bargaining and discussion with employees when an organizational change occurs).

While studies have demonstrated that some VOP are positively associated with indicators of well-being [20,21,22,23], to date, no work has tested the relationships between the joint effects of VOP, as an integrative construct, and psychological well-being, or identified the mechanisms underlying these relationships. Hence, the first aim of the present study was to identify the relationship between virtuous organizational practices and three indicators of well-being: job satisfaction (i.e., hedonic well-being), thriving at work (i.e., hedonic and eudaimonic well-being), and work–life balance (i.e., social well-being). The second aim was to test the mediational role of psychological capital in these relationships.

In brief, this study makes theoretical contributions to the scientific literature because it examines for the first time the links between a set of health-promoting organizational practices, based on the APA model of Psychologically healthy workplaces, and indicators of hedonic, eudaimonic, and social well-being, and goes beyond a simple description of these relationships by identifying a mechanism likely to mediate them. Moreover, there have been few studies on the effects of organizational practices on workers’ health, using the VOP construct. This study, therefore, aims to fill this gap by providing a better understanding of the effects of VOP on the French working population. Finally, the results of this study are promising for professionals, as VOP can be easily implemented and optimized in work organizations.

### 1.1. Virtuous Organizational Practices and Hedonic, Eudaimonic, and Social Well-Being

VOP are organizational practices that promote employees’ psychological health [24]. Thus, according to the Job Demands-Resources model (JD-R) [25,26], they can be considered as organizational resources. This model postulates that resources in the work environment generate a salutogenic process that strengthens workers’ health [27]. This is in line with the tenet of resource caravan passageways in the Conservation of Resources (COR) theory [28,29,30].

Hobfoll [29] explained that the individual resources of workers are likely to emerge and develop when they work in favorable social conditions, also called the *resource caravan passageways*. These are the environmental conditions of organizations that foster and protect the resources of staff; if individuals are able to develop and maintain their personal resources, the work environment has the power to support and enrich them. Thus, *resource caravan passageways* are environmental resources that might develop employees’ personal resources, in a “gain spiral”.

The positive impact of certain organizational resources on various dimensions of workers’ psychological health has been shown in a number of studies [31,32]. In the present study, VOP are, thus, considered as organizational resources that could contribute to the salutogenic process of the JD-R model [25,26] and as *resource caravan passageways* that could engender a virtuous process (i.e., a gain spiral) for the well-being of individuals [28,29]. Although there is no consensus on the definition of well-being [33], the one that is most commonly cited is the one proposed by the World Health Organization, namely “a state of complete physical, mental and social well-being” [34]. Moreover, authors agree that it is a multidimensional construct that includes hedonic, eudaimonic, and social well-being [35,36,37].

Hedonic well-being refers to the experience of pleasure and the avoidance of pain. It is characterized by the prevalence of positive affect (e.g., pleasure and joy) over negative affect (e.g., sadness), and by positive cognitive evaluations such as life or job satisfaction [37]. The latter is one of the most extensively studied indicators of well-being in the scientific literature [38] and is defined as “a pleasurable or positive emotional state resulting from the appraisal of one’s job or job experiences” (p. 1304) [39]. Several studies have demonstrated a positive relationship between some virtuous organizational practices and job satisfaction. For example, in a meta-analysis of 83 studies, authors [23] found that internal promotion, training, reward systems, and practices of participative decision-making, information sharing, and work–life balance were positively associated with hedonic well-being. Other researchers have observed that health, safety, psychosocial safety [40,41,42], and industrial relations climate [43] also promote job satisfaction. The results are in line with JD-R because they reveal that these practices are environmental resources that can promote psychological well-being (i.e., job satisfaction). In line with these studies, we formulated the following hypothesis:

**Hypothesis** **1.** *VOP are positively associated with job satisfaction*.

Eudaimonic well-being, or eudaimonia, refers to the development of human potential and optimal functioning [37]. This is expressed through striving to achieve excellence, virtue, and meaning in life. Although hedonic and eudaimonic well-being differ in their specific aims, namely, “feeling good” for the former, and development and self-realization for the latter, they are related [44], and they are both involved in thriving at work, which has been the subject of growing scientific interest in the international scientific community [45,46,47]. Thriving at work is characterized by a joint sense of vitality and of learning at work [48]. In other words, it is a psychological state with an affective and hedonic dimension (i.e., vitality), and a cognitive and eudaimonic dimension (i.e., learning). Workers who thrive, thus, feel energized and are enthusiastic and passionate about their work, and they have a sense of continually acquiring and applying knowledge [49].

Few studies to date have investigated the organizational antecedents of thriving at work, although its relationship with various positive behaviors and attitudes has been clearly recognized at both the individual and organizational level (e.g., work engagement, organizational citizenship behaviors, and creativity) [46]. For example, in line with the JD-R model [25,26], which postulates that organizational resources stimulate workers’ personal growth and development, Spreitzer et al. [48] argued that when employees are encouraged to participate in decision making (e.g., drawing up aims) and information sharing (e.g., about the functioning of their company), they are more likely to feel autonomous and free. This in turn boosts their adoption of agentic behaviors (e.g., exploring new alternatives) and optimizes their ability to seek relevant solutions to problems and make the best decisions, and to act quickly and effectively in difficult situations, ultimately helping them thrive at work. These hypotheses were confirmed by Zhang et al. [50] who tested the relationship between supervisors’ perceptions of High-Performance Work Systems, including training and performance appraisal, and employees’ thriving at work. More recently, Alikaj et al. [51] observed a positive correlation between employees’ perceptions of another set of organizational practices (i.e., High-Involvement HR Practices), including information sharing and incentive compensation, and their thriving at work. Thus, according to JD-R, these practices will generate work resources for employees, enabling them to thrive at work. In line with these findings, we formulated the following hypothesis:

**Hypothesis** **2.** *VOP are positively associated with thriving at work*.

Social well-being refers to how individuals perceive their circumstances and functioning in society [52]. It has recently been shown to be associated with indicators such as social harmony [53] and work–life balance [54]. The latter concerns “individuals’ perceptions of how well work and non-work roles fit together and are managed in accordance with their personal system of life values, goals, and aspirations” (p. 263) [54].

Some organizational practices are resources that enable employees to satisfy their work demands and their personal responsibilities more easily [55]. The first published works on the subject focused specifically on identifying organizational practices providing instrumental support to employees (e.g., flexible schedules and financial assistance), enabling them to meet the demands of their different life spheres and reduce their work–family conflicts [20,56,57]. There is a vast literature demonstrating a negative link between some organizational practices (e.g., schedule flexibility, on-site child care, and elder care assistance) and work–family conflict [20,58]. Recently, other studies have also demonstrated that some organizational practices in High-Performance Work systems (e.g., promotion, extensive training programs, and results-oriented appraisals) [55], high-involvement work processes (e.g., information sharing and rewards) [59], and psychosocial safety climate [60] are negatively associated with work–life imbalance and work–family conflict. However, studies on the relationships between organizational practices and work–life balance are still scarce [55,61] and have mainly focused on the positive role of work–life balance practices. For example, Choi et al. [62] demonstrated that family-supportive paid leave is positively associated with employees’ satisfaction with work–family balance. Regarding the VOP construct, while it includes practices that aim to protect this work–life balance (i.e., Work–Life Balance Practices), it also refers to practices that help workers better understand organizational processes (i.e., Communication Practices), defend their interests (i.e., Social Dialogue Practices), give their opinion on decisions that concern them (i.e., Practices of Participative Decision-Making), and obtain rewards (i.e., Recognition Practices). According to the JD-R model, VOP could, thus, provide staff with resources (e.g., information, freedom, and tangible support) to help them meet the demands of their professional and personal lives. Accordingly, we formulated the following hypothesis:

**Hypothesis** **3.** *VOP are positively associated with work–life balance*.

### 1.2. The Mediational Role of Psychological Capital

According to the COR theory [28,29], the professional context plays a role in the maintenance and development of employees’ resources, which, in turn, enhance their psychological health at work. More precisely, the COR theory [28,29] states that while workers strive to develop and maintain their personal resources, the work environment has the power to support or hinder them. For example, if the organization provides a sufficiently supportive environment, it will ensure the protection and even expansion of their employees’ resources, creating a gain spiral. Brummelhuis and Bakker [63] further postulated that there are “key resources” that facilitate the development of other resources such as self-efficacy and optimism [64]. The latter are sub-dimensions of Psychological Capital (PsyCap), which is defined as “an individual’s positive psychological state of development that is characterized by: (1) having confidence (self-efficacy) to take on and put in the necessary effort to succeed at challenging tasks; (2) making a positive attribution (optimism) about succeeding now and in the future; (3) persevering toward goals and, when necessary, redirecting paths to goals (hope) in order to succeed; and (4) when beset by problems and adversity, sustaining and bouncing back and even beyond (resilience) to attain success” (p. 334) [65]. The combination of self-efficacy, optimism, hope, and resilience generates a synergistic effect that can be captured through a second-order factor (i.e., psychological capital) [66].

Luthans et al. [65] defined the four resources of psychological capital (self-efficacy, optimism, hope, and resilience) as “state-like”. In other words, these resources are more stable than states, but more malleable than personal traits. For example, he explained that “optimism is created, motivated, and developed in relation to the pursuit of personally valuable goals” (p. 331) and can evolve over the long term. Thus, we chose the mediating role of psychological capital between VOP and the three indicators of well-being because it consists of four state-like resources and is, therefore, open to development. However, while these four personal resources are state-like and, thus, open to development [65], paradoxically, only a few studies have focused on their antecedents, although Avey [67] stressed the need to identify organizational policies and human resource management systems that support them. In our study, we hypothesized that VOP could be antecedents of PsyCap. First, some VOP (e.g., recognition practices and career management) could convince individuals that they possess the abilities and skills to succeed (mastery experiences and social persuasion) and, consequently, increase their feeling of self-efficacy in their chances of success [68]. Secondly, the JD-R model [25,26] postulates that resources, including organizational characteristics (i.e., such as VOP), can facilitate the achievement of work goals (hope). Indeed, organizations that encourage fair information sharing (e.g., communication practices and organizational justice) and clear decision-making procedures (e.g., organizational justice and participation in decision-making) allow workers to better understand organizational processes and identify what the organization expects of them in their work tasks and goals [65,69]. Thirdly, because organizational practices send a message to workers about management’s intentions and priorities [70], namely to promote their well-being, they can satisfy their positive expectations and increase their optimism. Finally, Masten and Reed [71] explained that clear organizational communication and participative decision-making practices enhance the individual’s capacity to bounce back after difficult events (i.e., resilience). More recently, Bardoel et al. [72] published a literature review in which they identified a set of human resource practices that can enhance employees’ resilience, including work–life balance practices, employee development programs, reward and benefits systems, and occupational health and safety systems.

More studies have investigated the consequences of PsyCap in the work sphere than its organizational antecedents [73,74]. In line with the COR theory [28,29] and the Broaden-and-Build Theory of Positive Emotions [75], Youssef-Morgan and Luthans [76] observed that PsyCap generates positive states that expand the thought–action repertoires of individuals and, thus, support the development of their physical, psychological, and social resources such as well-being. They argued that well-being is induced by positive cognitive and affective evaluations of past, present, and future events. Given that PsyCap refers to the “positive appraisal of circumstances and probability for success” (p. 550) [66], it shapes the way employees interpret their professional situation, leading to positive states such as job satisfaction [39,77]. Likewise, the meta-analyses of Avey et al. [73] and Kong et al. [74] revealed a strong correlation between PsyCap and job satisfaction. Moreover, Kleine et al. [46] postulated that employees are more likely to thrive when they have high PsyCap, which induces greater confidence and persistence in the achievement of their aims and in the development of action plans (self-efficacy and hope), more positive expectations (optimism), and a greater ability to bounce back and achieve success when faced with adversity (resilience). This was supported by the results of their meta-analysis with a total of 3985 employees [46], which revealed a positive relationship between PsyCap and thriving at work. Finally, several studies have shown that some individual resources of PsyCap reduce work–family imbalance; for example, the meta-analysis of Allen et al. [78] demonstrated that self-efficacy and optimism are negatively associated with work–family conflict. However, studies on the relationship between PsyCap and work–life balance remain scare [14,79].

More broadly, a number of multilevel studies have observed the mediating role of PsyCap in the relationship between the perceptions of managers and HR actors of certain organizational practices (e.g., High-Performance Work System and High-Commitment Work System) on the one hand, and indicators of employees’ attitudes and behavior at work on the other [55,80,81]. However, few studies have focused specifically on the mediating role of PsyCap in the relationship between employees’ perceptions of organizational practices such as VOP that foster their psychological health, and indicators of their hedonic, eudaimonic, and social well-being. The results are in line with JD-R and COR theory because they reveal that these practices are environmental resources that could develop employees’ resources (i.e., Psycap), which would, in turn, promote their psychological health, in a gain spiral. We, therefore, formulated the following hypotheses:

**Hypothesis** **4.** *PsyCap mediates the relationship between VOP and job satisfaction*.

**Hypothesis** **5.** *PsyCap mediates the relationship between VOP and thriving at work*.

**Hypothesis** **6.** *PsyCap mediates the relationship between VOP and work–life balance*.

## 2. Method

### 2.1. Procedure

A questionnaire was distributed to employees working in different sectors (non-profit, private, and public). The study was conducted in accordance with the Helsinki Declaration on Research Involving Human Beings [82], which guarantees anonymity, confidentiality, and informed consent. The research protocol was validated by the Tours-Poitiers Ethics Committee for Research, which is part of the University of Tours (CER-TP, file n° 2019-09-04).

### 2.2. Participants

In total, the sample consisted of 400 French workers, with 332 women (83.00%) and 68 men (17.00%). Their average age was 40.17 years (SD = 10.90) and the average tenure in their current employment was 8.51 years (SD = 8.74). Regarding their employment status, the majority of participants (84.25%) had a permanent contract (*n* = 337); 76.75% (*n* = 307) worked full-time and 23.00% (*n* = 92) worked part-time (1 person did not provide this information). Among the participants, 41.50% (*n* = 166) worked in the non-profit sector, 41.25% (*n* = 165) in the private sector, and 17.25% (*n* = 69) in the public sector. Finally, the average number of people employed in the organizations in which the participants worked was 154.67 (SD = 147.61), and the average number per department was 21.94 (SD = 28.88).

### 2.3. Measures

Participants completed the following scales using a five-point Likert scale ranging from 1 (“Strongly Disagree”) to 5 (“Strongly Agree”).

Virtuous organizational practices: VOP were assessed with the Virtuous Organizational Practices inventory (VOPi) [17]. This tool has 24 items divided into 8 sub-dimensions: practices of participative decision-making (e.g., “In my organization, employees are systematically consulted before important decisions are made”), work–life balance practices (e.g., “My organization offers everyone the possibility of organizing their own working time”), health and safety practices (e.g., “My organization supports preventive actions to foster its employees’ health”), recognition practices (e.g., “My organization recognizes the contribution of each individual to the effective functioning of the group”), practices of career management (e.g., “My organization supports the continuing education of all its employees”), communication practices (e.g., “My organization has different ways of disseminating information to all levels of the hierarchy”), organizational justice practices (e.g., “In my organization, the rules are applied to everyone in the same way”), and social dialogue practices (e.g., “My organization and the staff representatives work together to improve working conditions”). This scale demonstrated good internal consistency with an alpha of 0.94.

Psychological capital: PsyCap was measured using the short version of the Psychological Capital Questionnaire (PCQ) [83]. This scale has 12 items, including 4 for hope (e.g., “If I should find myself in a jam at work, I could think of many ways to get out of it”), 3 for self-efficacy (e.g., “I feel confident in representing my work area in meetings with management”), 3 for resilience (e.g., “I can get through difficult times at work because I’ve experienced difficulty before”), and 2 for optimism (e.g., “I’m optimistic about what will happen to me in the future as it pertains to work”). The internal consistency of this scale was satisfactory with an alpha of 0.82.

Job satisfaction: Job satisfaction was assessed with a one-item measure (i.e., “Overall, I am satisfied with my job”) [84].

Thriving at work: This was measured with Porath et al.’s [49] scale, which has 10 items: 5 for vitality (e.g., “I feel alive and vital”) and 5 for learning (e.g., “I continue to learn more as time goes by”). One item per sub-dimension was reversed (i.e., “I do not feel very energetic” and “I am not learning”). The internal consistency of this scale was satisfactory with an alpha of 0.84.

Work-life balance: This was measured with Haar’s [85] 3-item scale (e.g., “I manage to balance the demands of my work and personal/family life well”). The internal consistency of this scale was satisfactory with an alpha of 0.81.

## 3. Results and Discussion

### 3.1. Preliminary Analysis

Following Tabachnick and Fidell’s recommendations [86], 11 univariate outliers (i.e., |z| > 3.29, *p* < 0.001) and 9 multivariate outliers (i.e., Mahalanobis distance greater than χ^2^(16) = 39.25, *p* < 0.001) were excluded, leaving 380 participants for the analyses. All kurtosis and skewness coefficients were correct with values less than 10 for all components [87,88] (Table 1). For VOP, the mean score for all dimensions was 3.26 (Min = 2.76; Max = 3.51). For the other scales, mean scores were 3.69 for PsyCap, 3.80 for thriving at work, 3.96 for job satisfaction, and 3.66 for work–life balance. The data for VOP, PsyCap, thriving at work, job satisfaction, and work–life balance were normally distributed.

### 3.2. Validation of Measurement Models

In accordance with Anderson and Gerbing’s [89] recommendations, the measurement models were validated using confirmatory factor analysis (CFA) before testing the hypotheses using structural equation modeling (SEM). In order to determine the fit of the models to the data, we used the Maximum Likelihood Estimation (MLE), chi-square [90], Standardized Root-Mean-Square Residual (SRMR) [91], Root-Mean-Square Error of Approximation (RMSEA) [92], Comparative Fit Index (CFI) [93], and Akaike Information Criterion (AIC) [94].

Regarding VOP, the analyses revealed a good fit of the second-order structure model to the data (χ^2^ = 575.82 (244), *p* < 0.001; SRMR = 0.05; RMSEA = 0.06; CFI = 0.92; AIC = 735.82). A good fit was also found for second-order models tested for PsyCap (χ^2^ = 103.42 (49), *p* < 0.001; SRMR = 0.05; RMSEA = 0.05; CFI = 0.95; AIC = 185.42) and thriving at work (χ^2^ = 106.64 (29), *p* < 0.001; SRMR = 0.05; RMSEA = 0.08; CFI = 0.95; AIC = 178.64).

### 3.3. Correlation Analysis

The results of the correlation analyses (Table 2) revealed that virtuous organizational practices were positively and significantly correlated with job satisfaction (*r* = 0.40, *p* < 0.001), thriving at work (*r* = 0.48, *p* < 0.001), and work–life balance (*r* = 0.37, *p* < 0.001). Analyses also indicated positive and significant correlations between psychological capital on the one hand, and virtuous organizational practices (*r* = 0.32, *p* < 0.001), job satisfaction (*r* = 0.60, *p* < 0.001), thriving at work (*r* = 0.51, *p* < 0.001), and work–life balance (*r* = 0.57, *p* < 0.001) on the other. These results provide preliminary support for our hypotheses. Furthermore, the analyses revealed significant and positive correlations between job satisfaction and thriving at work (*r* = 0.51, *p* < 0.001), between job satisfaction and work–life balance (*r* = 0.46, *p* < 0.001), and between thriving at work and work–life balance (*r* = 0.46, *p* < 0.001).

### 3.4. Structural Equation Modeling

Next, we conducted structural equation modeling (SEM) using AMOS version 25 followed by a bootstrap analysis [95] to analyze the relationships between virtuous organizational practices on the one hand and job satisfaction, thriving at work, and work–life balance on the other, and to test the mediating role of PsyCap. To determine the fit of the models to the data, we used the fit indices mentioned above, together with the χ^2^/df ratio [88].

**Figure 1:**
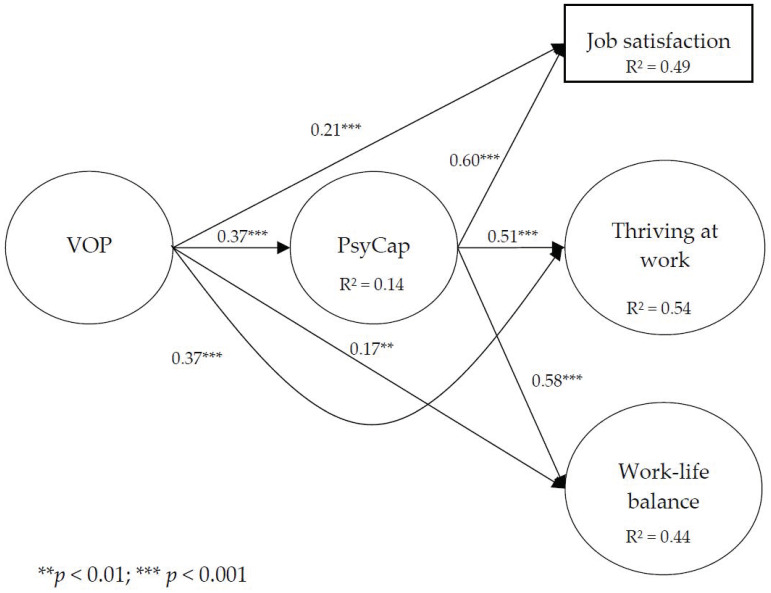
Results of the Hypothesized Model.

The first model included four latent variables and fifty observed variables. More specifically, relationships were identified between virtuous organizational practices, psychological capital, job satisfaction, thriving at work, and work–life balance. Because the sample was predominantly composed of women, the gender variable was controlled. Moreover, because some studies demonstrated differences in perception of organizational practices between sectors [96], the sector variable was also controlled. The results of the structural equation modeling showed satisfactory fit indices (χ^2^ = 2062.25 (1191), *p* < 0.001; SRMR = 0.06; RMSEA = 0.04; CFI = 0.90; AIC = 2434.25; χ^2^/df = 1.73). Analyses revealed that virtuous organizational practices were significantly and positively related to job satisfaction (β = 0.21, *p* < 0.001), thriving at work (β = 0.37, *p* < 0.001), work–life balance (β = 0.17, *p* < 0.01), and PsyCap (β = 0.37, *p* < 0.001), and that PsyCap was significantly and positively related to job satisfaction (β = 0.60, *p* < 0.001), thriving at work (β = 0.51, *p* < 0.001), and work–life balance (β = 0.58, *p* < 0.001) (Figure 1). In addition, the results suggested indirect effects of virtuous organizational practices on job satisfaction, thriving at work, and work–life balance through PsyCap.

This model was then compared to a second model in which PsyCap completely mediated these relationships (χ^2^ = 2125.48 (1194), *p* < 0.001; SRMR = 0.07; RMSEA = 0.05; CFI = 0.89; AIC = 2491.48; χ^2^/df = 1.78). The chi-square difference test detected a significant difference between the two models (Δχ^2^ = 63.23 (3), *p* < 0.001), indicating that the model with partial mediations fit the data better than the model with total mediations.

Finally, resampling analyses using the Bootstrap Confidence Interval [95] were conducted to assess the magnitude and significance of standardized indirect effects of virtuous organizational practices on the three psychological health indicators through PsyCap. Five thousand samples were, thus, generated from the original sample to calculate a confidence interval (95%) using the bootstrap percentile approach [95]. Results confirmed the indirect effects of virtuous organizational practices on job satisfaction (β = 0.22, SE = 0.05; CI = [0.14 to 0.32]; *p* < 0.001), thriving at work (β = 0.19, SE = 0.04; CI = [0.11 to 0.28]; *p* < 0.001), and work-life balance (β = 0.22, SE = 0.05; CI = [0.13 to 0.31]; *p* < 0.001) through PsyCap. The results indicated that psychological capital partially mediated the relationships between virtuous organizational practices and the well-being indicators studied.

## 4. Discussion

The aim of this study was to analyze the relationships between virtuous organizational practices and employees’ psychological well-being (i.e., hedonic, eudaimonic, and social) and to test the mediating role of psychological capital in these relationships.

First, the results confirm that virtuous organizational practices have direct and significant effects on the three dimensions of psychological well-being, namely job satisfaction (i.e., hedonic well-being), thriving at work (i.e., hedonic and eudaimonic well-being), and work–life balance (i.e., social well-being), thus validating hypotheses 1, 2, and 3. These results are innovative because they provide evidence of the beneficial effects of virtuous organizational practices on employees’ well-being, over and above commonly used indicators of well-being. Indeed, in this research field, thriving at work and work–life balance are rarely investigated. Consequently, the results also clearly confirm the relevance of the VOP integrative construct (i.e., organizational practices promoting optimal health ) [17] and its additional heuristic power, because it enables researchers to examine the joint effect of many organizational practices on various indicators of well-being. Moreover, this study also demonstrates that VOP can be considered as *resource caravan passageways* as described in the COR theory (i.e., “environmental conditions that support, foster, enrich, and protect the resources of individuals, sections or segments of workers, and organizations in total”; p. 118) and, thus, have the capacity to protect and develop workers’ individual resources [29].

Secondly, our results reveal that psychological capital partially mediates the relationships between VOP and the three selected well-being indicators, thus validating hypotheses 4, 5, and 6. In this way, our findings contribute to knowledge of the psychological mechanisms underlying the effects of virtuous organizational practices on workers’ psychological health. Indeed, while the COR theory [29] assumes that the work environment can support workers’ personal resources and, thus, foster a virtuous spiral, our research clarifies one of the psychological mechanisms (i.e., psychological capital) underlying this chain of relationships. Specifically, the perception that their organization implements VOP is likely to increase employees’ sense of self-efficacy, hope, optimism, and resilience, which, in turn, could promote their hedonic, eudaimonic, and social well-being. Thus, contextual resources of the organization (i.e., VOP) are conducive to the development of their employees’ personal resources (i.e., psychological capital), which, in turn, are likely to generate other individual resources (i.e., job satisfaction, thriving at work, and work–life balance). These results are consistent with the notion of “resource caravans” [28,29], which postulates that personal resources are interdependent and enable the acquisition and protection of other resources. The results also support Youssef-Morgan and Luthans’ [76] hypothesis that positive states generated by PsyCap are likely to expand workers’ thinking and acting and produce new physical, psychological, and social resources. In this way, psychological capital could refer to what ten Brummelhuis and Bakker [63] called “key resources”; in other words, those that facilitate the development of other resources. Finally, this study indicates that virtuous organizational practices could be considered as one of the determinants of PsyCap, adding to the literature on its antecedents, which are more rarely examined than its consequences [67].

## 5. Limitations and Future Research

First, although our hypotheses were based on the scientific literature, our cross-sectional design makes it impossible to draw conclusions about the causal link between the variables studied. Future studies should, thus, explore the issue of directionality through longitudinal and/or experimental designs to clearly establish causal relationships between virtuous organizational practices and psychological well-being.

Secondly, even though the psychometric qualities of the scales used in this study were carefully checked, another limitation concerns the use of self-report questionnaires, which increases the risk of social desirability and the common-method bias. According to Podsakoff et al. [97], these are the main sources of measurement error and threaten the validity of the conclusions. To overcome this problem and further our understanding of the effects of VOP, future studies could combine perceptual measures with objective measures of health.

A third limitation concerns the characteristics of our convenience sample. Eighty-three percent of the sample were female, and we, therefore, controlled for gender in the analyses. However, previous studies have observed that gender moderates the relationship between organizational practices and attitudinal and behavioral consequences at work. For example, Andersén and Andersén [98] and Shin et al. [99] showed that the influence of organizational practices (i.e., High-Performance Work Systems and Human Resource Management) on employees’ affective commitment was significantly greater for women than for men. Further research grounded on a representative sample of the male–female distribution in the French worker population is, thus, needed to contribute to this field of study. Moreover, as the meta-analysis by Blom et al. [96] found significant differences in HRM between different sectors (public, semi-public, and private), future research could compare employees’ perceptions of VOP by sector.

A fourth limitation is related to the consequences of VOP and the psychological mechanisms studied in this research. Indeed, these are exclusively indicators of well-being and personal resources. There are virtually no studies that have tested the joint effects of VOP on employee attitudes and behaviors. More precisely, while some studies demonstrated that these practices considered in isolation have a positive effect on these attitudinal and behavioral consequences [23,100], to our knowledge, their joint effects have only been tested once. While Aubouin-Bonnaventure et al. [101] recently found that VOP are predictors of employees’ organizational citizenship behavior and intention to remain, no study has yet identified the relationships between VOP and employees’ positive attitudes (e.g., organizational commitment) and behaviors (e.g., prosocial behaviors). Moreover, although the analyses reveal that psychological capital underlies the effect of virtuous organizational practices on workers’ well-being, it only emerged as a partial mediator of these relationships. These results, thus, suggest that future research should identify other underlying mechanisms.

Finally, this study did not take line management into consideration (e.g., direct leaders and HR actors). However, studies have revealed the crucial role of direct leaders’ attitudes and communication in the perception and use of organizational practices by their subordinates [102]. Indeed, direct leaders are the most proximal representatives of the organization and are “gate-keepers” for everything that occurs in the organization [103]. Thus, future research should consider the role of the managerial line on the effect of VOP on employee health.

### Practical Implications

From a practical point of view, this study provides a concrete response to a crucial need of work organizations, namely to protect and promote the psychological well-being of their personnel. Indeed, psychological health is at the heart of current concerns, particularly during and following the COVID-19 pandemic [104], because it involves multiple issues at the individual, group, and organizational levels. For example, when the work fosters the well-being of employees, they are less exhausted [105], are less absent [106], and have less intention to leave the organization [46]. Therefore, workers’ psychological well-being represents a lever of staff retention for organizations. Furthermore, employees who are fulfilled at work are also more creative [46] and engage in more innovative behaviors [107], workplace civility, supportive coworker behaviors, and heedful relating [46]. In brief, improving the optimal functioning of their workers ensures the organization’s sustainability.

The results of this study show that VOP offer a promising avenue for action in work organizations; they demonstrate that by implementing or optimizing VOP, organizations can increase the psychological capital of their employees over time and, thus, their psychological well-being. VOP should, thus, be thought of and incorporated in the organization’s strategic policies. Their implementation and optimization should be primary prevention actions. Concretely, employers could ask their staff to complete the VOPi (i.e., Virtuous Organizational Practices inventory) [17] in order to identify the organizational practices perceived to be lacking or inadequate and introduce appropriate actions. For example, if employees perceive work–life balance practices to be lacking, management could provide additional resources to meet the demands of their different life roles by introducing more flexible work schedules, part-time work, telecommuting, or tangible support such as financial aid (e.g., subsidies for childcare) [108,109]. Finally, managers should be made aware of the importance of communication with their subordinates on practices and should be trained in managerial behaviors in line with VOP, such as R.I.G.H.T leadership [110] or positive leadership [111].

## 6. Conclusions

The results of this study are promising because virtuous organizational practices can easily be implemented and optimized in work organizations to develop the individual resources of workers, and ultimately to promote their psychological well-being. These practices are, therefore, a crucial strategy to protect the employees’ psychological health and to ensure the organizations’ sustainability.

## Figures and Tables

**Table 1:** Descriptive statistics for the study variables.

	M	SD	Skewness Coefficients	Kurtosis Coefficients	α
VOP	3.26	0.57	−0.34	0.94	0.93
PPDM	2.76	0.83	0.00	−0.36	
WLBP	2.94	0.78	−0.19	0.04	
HSP	3.49	0.70	−0.55	0.59	
RP	3.29	0.79	−0.31	−0.09	
PCM	3.51	0.72	−0.55	0.53	
CP	3.49	0.74	−0.85	0.81	
OJP	3.28	0.74	−0.20	0.29	
SDP	3.33	0.68	−0.44	0.99	
PsyCap	3.69	0.43	−0.12	0.58	0.82
Hope	3.74	0.48	−0.23	0.75	
Self-efficacy	3.79	0.57	−0.27	0.65	
Resilience	3.74	0.53	−0.01	0.28	
Optimism	3.49	0.66	−0.26	−0.21	
Job satisfaction	3.96	0.67	−0.75	1.43	-
Thriving at work	3.80	0.52	−0.50	0.87	0.86
Vitality	3.85	0.60	−0.74	1.29	
Learning	3.75	0.54	−0.33	0.56	
Work-life balance	3.66	0.72	−0.49	0.22	0.80

Note. VOP = Virtuous Organizational Practices; PPDM = Practices of Participative Decision-Making; WLBP = Work–Life Balance Practices; HSP = Health and Safety Practices; RP = Recognition Practices; PCM = Practices of Career Management; CP = Communication Practices; OJP = Organizational Justice Practices; SDP = Social Dialogue Practices; PsyCap = Psychological Capital.

**Table 2:** Correlations for the study variables.

	1	2	3	4	5
1 VOP	_				
2 PsyCap	0.32	_			
3 Job satisfaction	0.40	0.60	_		
4 Thriving at work	0.48	0.51	0.51	_	
5 Work-life balance	0.37	0.57	0.46	0.46	_

All correlations are significant at *p* < 0.001. Note. VOP = Virtuous Organizational Practices; PsyCap = Psychological Capital.

## Data Availability

Material and datasets generated for this study are available on request to the corresponding author.

## References

[1] Allan B.A., Kim T., Liu T.Y., Owens R.L. (2021). Are work well-being variables distinct? A bifactor model of fulfilling work. J. Couns. Psychol..

[2] Ilies R., Wagner D., Wilson K., Ceja L., Johnson M., Derue S., Ilgen D. (2017). Flow at Work and Basic Psychological Needs: Effects on Well-Being. Appl. Psychol..

[3] Alikaj Y., Xu S., Jin J., Ford M.T. (2018). The within and cross domain effects of work-family enrichment: A meta-analysis. J. Vocat. Behav..

[4] Blanco-Encomienda F.J., García-Cantero R., Latorre-Medina M.J. (2020). Association between Work-Related Rumination, Work Environment and Employee Well-Being: A Meta-Analytic Study of Main and Moderator Effects. Soc. Indic. Res..

[5] Fasbender U., Van Der Heijden B.I.J.M., Grimshaw S. (2019). Job satisfaction, job stress and nurses’ turnover intentions: The moderating roles of on-the-job and off-the-job embeddedness. J. Adv. Nurs..

[6] Khamisa N., Peltzer K., Ilic D., Oldenburg B. (2016). Work related stress, burnout, job satisfaction and general health of nurses: A follow-up study. Int. J. Nurs. Pract..

[7] Ford M.T., Cerasoli C.P., Higgins J.A., DeCesare A.L. (2011). Relationships between psychological, physical, and behavioural health and work performance: A review and meta-analysis. Work. Stress.

[8] Gutiérrez O.I., Polo J.D., Zambrano M.J., Molina D.C. (2020). Meta-analysis and Scientific Mapping of Well-being and Job Performance. Span. J. Psychol..

[9] Meyer J.P., Stanley D.J., Herscovitch L., Topolnytsky L. (2002). Affective, Continuance, and Normative Commitment to the Organization: A Meta-analysis of Antecedents, Correlates, and Consequences. J. Vocat. Behav..

[10] Eatough E.M., Chang C.-H., Miloslavic S.A., Johnson R.E. (2011). Relationships of role stressors with organizational citizenship behavior: A meta-analysis. J. Appl. Psychol..

[11] Dirks K.T., Ferrin D.L. (2002). Trust in leadership: Meta-analytic findings and implications for research and practice. J. Appl. Psychol..

[12] Nielsen K., Nielsen M.B., Ogbonnaya C., Känsälä M., Saari E., Isaksson K. (2017). Workplace resources to improve both employee well-being and performance: A systematic review and meta-analysis. Work. Stress.

[13] Chevalier S., Lejeune J., Fouquereau E., Coillot H., Gillet N., Gandemer V., Michon J., Colombat P. (2017). Organizational and Managerial Resources and Quality of Care in French Pediatric Oncology Nursing. J. Pediatr. Oncol. Nurs..

[14] Chevalier S., Coillot H., Colombat P., Bosselut G., Guilbert L., Fouquereau E. (2021). An explanatory model of authentic leadership, flourishing and work–family balance of nurses in French hospitals. Leadersh. Health Serv..

[15] Gillet N., Gagné M., Sauvagère S., Fouquereau E. (2013). The role of supervisor autonomy support, organizational support, and autonomous and controlled motivation in predicting employees’ satisfaction and turnover intentions. Eur. J. Work. Organ. Psychol..

[16] Su L., Swanson S.R. (2019). Perceived corporate social responsibility’s impact on the well-being and supportive green behaviors of hotel employees: The mediating role of the employee-corporate relationship. Tour. Manag..

[17] Aubouin-Bonnaventure J., Fouquereau E., Coillot H., Lahiani F.J., Chevalier S. (2021). Virtuous Organizational Practices: A New Construct and a New Inventory. Front. Psychol..

[18] Cameron K.S., Caza A. (2004). Introduction: Contributions to the Discipline of Positive Organizational Scholarship. Am. Behav. Sci..

[19] Meyer M. (2018). The Evolution and Challenges of the Concept of Organizational Virtuousness in Positive Organizational Scholarship. J. Bus. Ethics.

[20] Butts M.M., Casper W.J., Yang T.S. (2013). How important are work–family support policies? A meta-analytic investigation of their effects on employee outcomes. J. Appl. Psychol..

[21] Clarke S. (2010). An integrative model of safety climate: Linking psychological climate and work attitudes to individual safety outcomes using meta-analysis. J. Occup. Organ. Psychol..

[22] Grawitch M.J., Trares S., Kohler J.M. (2007). Healthy workplace practices and employee outcomes. Int. J. Stress Manag..

[23] Kooij D.T.A.M., Jansen P.G.W., Dikkers J.S.E., De Lange A.H. (2010). The influence of age on the associations between HR practices and both affective commitment and job satisfaction: A meta-analysis. J. Organ. Behav..

[24] Aubouin-Bonnaventure J., Chevalier S., Beltou N., Fouquereau E., Aguerre C., Chasseigne G. (2022). Comportements et Contextes Professionnels Positifs: Un Changement Majeur de Paradigme. Diversité et Actualité de la Psychologie Positive.

[25] Bakker A.B., Demerouti E. (2007). The Job Demands-Resources model: State of the art. J. Manag. Psychol..

[26] Demerouti E., Bakker A.B., Nachreiner F., Schaufeli W.B. (2001). The job demands-resources model of burnout. J. Appl. Psychol..

[27] Brauchli R., Jenny G.J., Füllemann D., Bauer G.F. (2015). Towards a Job Demands-Resources Health Model: Empirical Testing with Generalizable Indicators of Job Demands, Job Resources, and Comprehensive Health Outcomes. BioMed Res. Int..

[28] Hobfoll S.E. (1989). Conservation of resources: A new attempt at conceptualizing stress. Am. Psychol..

[29] Hobfoll S.E. (2011). Conservation of resource caravans and engaged settings: Conservation of resource caravans. J. Occup. Organ. Psychol..

[30] Hobfoll S.E., Halbesleben J., Neveu J.-P., Westman M. (2018). Conservation of Resources in the Organizational Context: The Reality of Resources and Their Consequences. Annu. Rev. Organ. Psychol. Organ. Behav..

[31] Caesens G., Stinglhamber F. (2014). The relationship between perceived organizational support and work engagement: The role of self-efficacy and its outcomes. Eur. Rev. Appl. Psychol..

[32] Wolter C., Maria A.S., Wörfel F., Gusy B., Lesener T., Kleiber D., Renneberg B. (2018). Job Demands, Job Resources, and Well-being in Police Officers—A Resource-Oriented Approach. J. Police Crim. Psychol..

[33] Danna K., Griffin R.W. (1999). Health and Well-Being in the Workplace: A Review and Synthesis of the Literature. J. Manag..

[34] World Health Organization Preamble of the Constitution of the World Health Organization as Adopted by the International Health Conference, New York, NY, USA, 19 June–22 July 1946; Signed on 22 July 1946 by the Representatives of 61 States (Official Record of the World Health Organization, no. 2, p. 100) and Entered into Force on 7 April 1948. http://whqlib-doc.who.int/hist/official_records/constitution.pdf.

[35] Keyes C.L.M. (2003). Complete mental health: An agenda for the 21st century. Flourishing: Positive Psychology and the Life Well-Lived.

[36] Keyes C.L.M. (2005). Mental Illness and/or Mental Health? Investigating Axioms of the Complete State Model of Health. J. Consult. Clin. Psychol..

[37] Ryan R.M., Deci E.L. (2001). On Happiness and Human Potentials: A Review of Research on Hedonic and Eudaimonic Well-Being. Annu. Rev. Psychol..

[38] Li M., Pérez-Díaz P.A., Mao Y., Petrides K.V. (2018). A Multilevel Model of Teachers’ Job Performance: Understanding the Effects of Trait Emotional Intelligence, Job Satisfaction, and Organizational Trust. Front. Psychol..

[39] Locke E.A., Dunnette M.D. (1976). The Nature and Causes of Job Satisfaction. Handbook of Industrial and Organizational Psychology.

[40] Geisler M., Berthelsen H., Muhonen T. (2019). Retaining Social Workers: The Role of Quality of Work and Psychosocial Safety Climate for Work Engagement, Job Satisfaction, and Organizational Commitment. Hum. Serv. Organ. Manag. Leadersh. Gov..

[41] Gragnano A., Miglioretti M., Frings-Dresen M.H.W., de Boer A.G.E.M. (2017). Adjustment between work demands and health needs: Development of the Work–Health Balance Questionnaire. Rehabil. Psychol..

[42] Nixon A.E., Lanz J.J., Manapragada A., Bruk-Lee V., Schantz A., Rodriguez J.F. (2015). Nurse safety: How is safety climate related to affect and attitude?. Work. Stress.

[43] Snape E., Redman T. (2012). Industrial Relations Climate and Union Commitment: An Evaluation of Workplace-Level Effects. Ind. Relat. J. Econ. Soc..

[44] Ryan R.M., Huta V., Deci E.L. (2008). Living well: A self-determination theory perspective on eudaimonia. J. Happiness Stud..

[45] Gerbasi A., Porath C.L., Parker A., Spreitzer G., Cross R. (2015). Destructive de-energizing relationships: How thriving buffers their effect on performance. J. Appl. Psychol..

[46] Kleine A., Rudolph C.W., Zacher H. (2019). Thriving at work: A meta-analysis. J. Organ. Behav..

[47] Prem R., Ohly S., Kubicek B., Korunka C. (2017). Thriving on challenge stressors? Exploring time pressure and learning demands as antecedents of thriving at work. J. Organ. Behav..

[48] Spreitzer G., Sutcliffe K., Dutton J., Sonenshein S., Grant A.M. (2005). A Socially Embedded Model of Thriving at Work. Organ. Sci..

[49] Porath C., Spreitzer G., Gibson C., Garnett F.G. (2012). Thriving at work: Toward its measurement, construct validation, and theoretical refinement. J. Organ. Behav..

[50] Zhang J., Bal P.M., Akhtar M.N., Long L., Zhang Y., Ma Z. (2018). High-performance work system and employee performance: The mediating roles of social exchange and thriving and the moderating effect of employee proactive personality. Asia Pac. J. Hum. Resour..

[51] Alikaj A., Ning W., Wu B. (2021). Proactive Personality and Creative Behavior: Examining the Role of Thriving at Work and High-Involvement HR Practices. J. Bus. Psychol..

[52] Keyes C.L.M. (1998). Social Well-Being. Soc. Psychol. Q..

[53] Gilbert M.-H., Dagenais-Desmarais V., Savoie A. (2011). Validation d’une mesure de santé psychologique au travail. Eur. Rev. Appl. Psychol..

[54] Haar J.M., Sune A., Russo M., Ollier-Malaterre A. (2019). A Cross-National Study on the Antecedents of Work–Life Balance from the Fit and Balance Perspective. Soc. Indic. Res..

[55] Wattoo M.A., Zhao S., Xi M. (2019). High-performance work systems and work–family interface: Job autonomy and self-efficacy as mediators. Asia Pac. J. Hum. Resour..

[56] Baltes B.B., Briggs T.E., Huff J.W., Wright J.A., Neuman G.A. (1999). Flexible and compressed workweek schedules: A meta-analysis of their effects on work-related criteria. J. Appl. Psychol..

[57] Kossek E.E., Pichler S., Bodner T., Hammer L.B. (2011). Workplace Social Support and Work-Family Conflict: A Meta-Analysis Clarifying the Influence of General and Work-Family-Specific Supervisor and Organizational Support. Pers. Psychol..

[58] Michel J.S., Kotrba L.M., Mitchelson J.K., Clark M.A., Baltes B.B. (2011). Antecedents of work-family conflict: A meta-analytic review. J. Organ. Behav..

[59] Boxall P., Macky K. (2014). High-involvement work processes, work intensification and employee well-being. Work. Employ. Soc..

[60] Mansour S., Tremblay D.-G. (2018). Psychosocial safety climate as resource passageways to alleviate work-family conflict: A study in the health sector in Quebec. Pers. Rev..

[61] Carvalho V.S., Chambel M.J. (2015). Perceived High-Performance Work Systems and Subjective Well-Being: Work-to-Family Balance and Well-Being at Work as Mediators. J. Career Dev..

[62] Choi J., Kim A., Han K., Ryu S., Park J.G., Kwon B. (2017). Antecedents and consequences of satisfaction with work-family balance: A moderating role of perceived insider status. J. Organ. Behav..

[63] Ten Brummelhuis L.L., Bakker A.B. (2012). A resource perspective on the work–home interface: The work–home resources model. Am. Psychol..

[64] Hobfoll S.E. (2002). Social and Psychological Resources and Adaptation. Rev. Gen. Psychol..

[65] Luthans F., Youssef C.M. (2007). Emerging Positive Organizational Behavior. J. Manag..

[66] Luthans F., Avolio B.J., Avey J.B., Norman S.M. (2007). Positive Psychological Capital: Measurement and Relationship with Performance and Satisfaction. Pers. Psychol..

[67] Avey J.B. (2014). The Left Side of Psychological Capital: New Evidence on the Antecedents of PsyCap. J. Leadersh. Organ. Stud..

[68] Bandura A., Ramachaudran V.S. (1994). Self-Efficacy. Encyclopedia of Human Behavior.

[69] Hur W.-M., Rhee S.-Y., Ahn K.-H. (2016). Positive psychological capital and emotional labor in Korea: The job demands-resources approach. Int. J. Hum. Resour. Manag..

[70] Van De Voorde K., Beijer S. (2014). The role of employee HR attributions in the relationship between high-performance work systems and employee outcomes. Hum. Resour. Manag. J..

[71] Masten A.S., Reed M.J., Snyder C.R., Lopez S.J. (2002). Resilience in development. Handbook of Positive Psychology.

[72] Bardoel E.A., Pettit T.M., De Cieri H., McMillan L. (2014). Employee resilience: An emerging challenge for HRM. Asia Pac. J. Hum. Resour..

[73] Avey J.B., Reichard R.J., Luthans F., Mhatre K.H. (2011). Meta-analysis of the impact of positive psychological capital on employee attitudes, behaviors, and performance. Hum. Resour. Dev. Q..

[74] Kong F., Tsai C.-H., Tsai F.-S., Huang W., De la Cruz S.M. (2018). Psychological Capital Research: A Meta-Analysis and Implications for Management Sustainability. Sustainability.

[75] Fredrickson B.L. (1998). What Good Are Positive Emotions?. Rev. Gen. Psychol..

[76] Youssef-Morgan C.M., Luthans F. (2015). Psychological Capital and Well-being. Stress Health.

[77] Fouquereau E., Rioux L. (2002). Élaboration de l’Échelle de satisfaction de vie professionnelle (ÉSVP) en langue française: Une démarche exploratoire. Can. J. Behav. Sci./Rev. Can. des Sci. du Comport..

[78] Allen T.D., Johnson R.C., Saboe K.N., Cho E., Dumani S., Evans S. (2012). Dispositional variables and work–family conflict: A meta-analysis. J. Vocat. Behav..

[79] Siu O.L. (2013). Psychological Capital, Work Well-Being, and Work-Life Balance Among Chinese Employees: A Cross-Lagged Analysis. J. Pers. Psychol..

[80] Chen S.-L. (2018). Cross-level effects of high-commitment work systems on work engagement: The mediating role of psychological capital. Asia Pac. J. Hum. Resour..

[81] Miao R., Bozionelos N., Zhou W., Newman A. (2021). High-performance work systems and key employee attitudes: The roles of psychological capital and an interactional justice climate. Int. J. Hum. Resour. Manag..

[82] World Medical Association (2013). World Medical Association Declaration of Helsinki: Ethical principles for medical research involving human subjects. JAMA.

[83] Lorenz T., Beer C., Pütz J., Heinitz K. (2016). Measuring Psychological Capital: Construction and Validation of the Compound PsyCap Scale (CPC-12). PLoS ONE.

[84] Tavani J., Botella M., Collange J. (2014). Quelle validité pour une mesure de la satisfaction au travail en un seul item?. Prat. Psychol..

[85] Haar J.M. (2013). Testing a new measure of work–life balance: A study of parent and non-parent employees from New Zealand. Int. J. Hum. Resour. Manag..

[86] Tabachnick B.G., Fidell L.S. (2013). Using Multivariate Statistics.

[87] George D., Mallery P. (2010). SPSS for Windows Step by Step: A Simple Guide and Reference.

[88] Kline R.B. (2016). Principles and Practice of Structural Equation Modeling.

[89] Anderson J.C., Gerbing D.W. (1988). Structural equation modeling in practice: A review and recommended two-step approach. Psychol. Bull..

[90] Jöreskog K.G. (1967). Some contributions to maximum likelihood factor analysis. Psychometrika.

[91] Jöreskog J., Sörbom D. (2001). LISREL 8.50.

[92] Browne M., Cudeck R. (1989). Single Sample Cross-Validation Indices for Covariance Structures. Multivar. Behav. Res..

[93] Bentler P.M. (1990). Comparative Fit Indexes in Structural Models. Psychol. Bull..

[94] Akaike H. (1987). Factor analysis and AIC. Psychometrika.

[95] Hayes A.F. (2018). Introduction to Mediation, Moderation, and Conditional Process Analysis: A Regression-Based Approach.

[96] Blom R., Kruyen P.M., Van Der Heijden B.I.J.M., Van Thiel S. (2020). One HRM Fits All? A Meta-Analysis of the Effects of HRM Practices in the Public, Semipublic, and Private Sector. Rev. Public Pers. Adm..

[97] Podsakoff P.M., MacKenzie S.B., Lee J.-Y., Podsakoff N.P. (2003). Common method biases in behavioral research: A critical review of the literature and recommended remedies. J. Appl. Psychol..

[98] Andersén J., Andersén A. (2019). Are high-performance work systems (HPWS) appreciated by everyone? The role of management position and gender on the relationship between HPWS and affective commitment. Empl. Relat. Int. J..

[99] Shin D., Garmendia A., Ali M., Konrad A.M., Madinabeitia-Olabarria D. (2020). HRM systems and employee affective commitment: The role of employee gender. Gend. Manag. Int. J..

[100] Colquitt J.A., Conlon D.E., Wesson M.J., Porter C.O.L.H., Ng K.Y. (2001). Justice at the millennium: A meta-analytic review of 25 years of organizational justice research. J. Appl. Psychol..

[101] Aubouin-Bonnaventure J., Chevalier S., Coillot H., Lahiani F.-J., Fouquereau E. (2022). Effets des pratiques organisationnelles vertueuses sur les comportements citoyens et l’intention de rester dans l’entreprise: Le rôle médiateur de l’adéquation personne-organisation. Psychol. du Trav. et des Organ..

[102] Sweet S., Pitt-Catsouphes M., James J.B. (2017). Manager attitudes concerning flexible work arrangements: Fixed or changeable?. Community Work. Fam..

[103] Zohar D., Luria G. (2010). Group Leaders as Gatekeepers: Testing Safety Climate Varia- tions across Levels of Analysis. Appl. Psychol..

[104] Meyer B., Zill A., Dilba D., Gerlach R., Schumann S. (2021). Employee psychological well-being during theCOVID-19 pandemic in Germany: A longitudinal study of demands, resources, and exhaustion. Int. J. Psychol..

[105] Wang W., Yin H., Huang S. (2016). The missing links between emotional job demand and exhaustion and satisfaction: Testing a moderated mediation model. J. Manag. Organ..

[106] Wegge J., Schmidt K.-H., Parkes C., Dick R. (2007). Taking a sickie: Job satisfaction and job involvement as interactive predictors of absenteeism in a public organization. J. Occup. Organ. Psychol..

[107] Tang Y., Shao Y.-F., Chen Y.-J. (2019). Assessing the Mediation Mechanism of Job Satisfaction and Organizational Commitment on Innovative Behavior: The Perspective of Psychological Capital. Front. Psychol..

[108] Bin Bae K., Goodman D. (2014). The Influence of Family-Friendly Policies on Turnover and Performance in South Korea. Public Pers. Manag..

[109] Hill E.J., Grzywacz J.G., Allen S., Blanchard V.L., Matz-Costa C., Shulkin S., Pitt-Catsouphes M. (2008). Defining and conceptualizing workplace flexibility. Community Work. Fam..

[110] Gulseren D.B., Thibault T., Kelloway E.K., Mullen J., Teed M., Gilbert S., Dimoff J.K. (2021). R.I.G.H.T. leadership: Scale development and validation of a psychologically healthy leadership model. Can. J. Adm. Sci./Rev. Can. des Sci. de l’Administration.

[111] Redín D.M., Meyer M., Rego A. (2023). Positive leadership action framework: Simply doing good and doing well. Front. Psychol..

